# Behavioural subphenotypes and their anatomic correlates in neurodegenerative disease

**DOI:** 10.1093/braincomms/fcad038

**Published:** 2023-02-27

**Authors:** Ashlin R K Roy, Samir Datta, Emily Hardy, Virginia E Sturm, Joel H Kramer, William W Seeley, Katherine P Rankin, Howard J Rosen, Bruce L Miller, David C Perry

**Affiliations:** Department of Neurology, University of California, San Francisco 94158, USA; Department of Neurology, University of California, San Francisco 94158, USA; Department of Neurology, University of California, San Francisco 94158, USA; Department of Neurology, University of California, San Francisco 94158, USA; Department of Psychiatry, University of California, San Francisco 94143, USA; Department of Neurology, University of California, San Francisco 94158, USA; Department of Neurology, University of California, San Francisco 94158, USA; Department of Neurology, University of California, San Francisco 94158, USA; Department of Neurology, University of California, San Francisco 94158, USA; Department of Neurology, University of California, San Francisco 94158, USA; Department of Neurology, University of California, San Francisco 94158, USA

**Keywords:** neurodegenerative diseases, dementia, Alzheimer’s disease, frontotemporal dementia, neuropsychiatric symptoms

## Abstract

Patients with neurodegenerative disorders experience a range of neuropsychiatric symptoms. The neural correlates have been explored for many individual symptoms, such as apathy and disinhibition. Atrophy patterns have also been associated with broadly recognized syndromes that bring together multiple symptoms, such as the behavioural variant of frontotemporal dementia. There is substantial heterogeneity of symptoms, with partial overlap of behaviour and affected neuroanatomy across and within dementia subtypes. It is not well established if there are anatomically distinct behavioural subphenotypes in neurodegenerative disease. The objective of this study was to identify shared behavioural profiles in frontotemporal dementia-spectrum and Alzheimer’s disease-related syndromes. Additionally, we sought to determine the underlying neural correlates of these symptom clusters. Two hundred and eighty-one patients diagnosed with one of seven different dementia syndromes, in addition to healthy controls and individuals with mild cognitive impairment, completed a 109-item assessment capturing the severity of a range of clinical behaviours. A principal component analysis captured distinct clusters of related behaviours. Voxel-based morphometry analyses were used to identify regions of volume loss associated with each component. Seven components were identified and interpreted as capturing the following behaviours: Component 1—emotional bluntness, 2—emotional lability and disinhibition, 3—neuroticism, 4—rigidity and impatience, 5—indiscriminate consumption, 6—psychosis and 7—Geschwind syndrome-related behaviours. Correlations with structural brain volume revealed distinct neuroanatomical patterns associated with each component, including after controlling for diagnosis, suggesting that localized neurodegeneration can lead to the development of behavioural symptom clusters across various dementia syndromes.

## Teaser

Patients with one of seven different dementia syndromes, mild cognitive impairment, or controls (*n* = 281), completed a 109-item assessment capturing the severity of a range of clinical behaviours. A principal component analysis identified seven components of related behaviours. Imaging analyses identified regions of volume loss associated with each component.

## Graphical Abstract

**Graphical Abstract:**
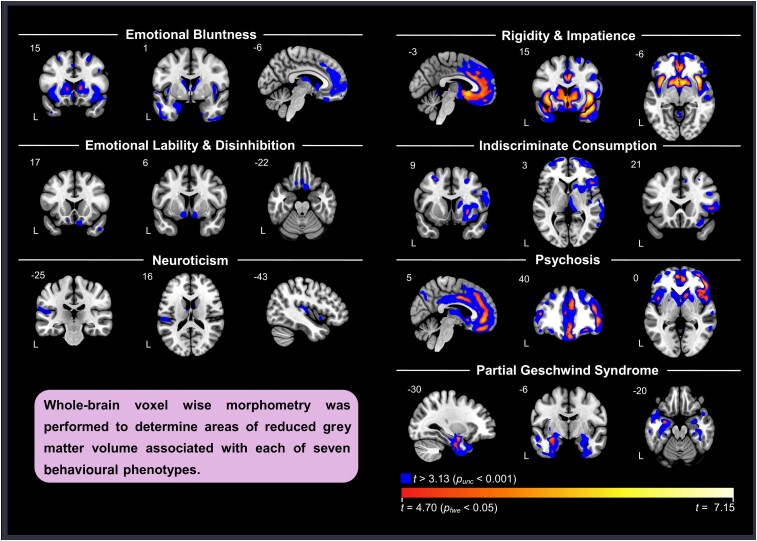

## Introduction

Symptom profiles in neurodegenerative disease vary depending on which anatomic regions are most vulnerable initially and throughout the disease course. While some neurodegenerative diseases, such as Alzheimer’s disease, are best known for causing cognitive dysfunction, and others cause characteristic motor impairment, including progressive supranuclear palsy, these illnesses also affect systems involved in behaviour. Neuropsychiatric symptoms commonly occur in these and a wide range of other neurodegenerative syndromes and are a significant driver of functional impairment and caregiver stress.^1–4^ Patients with Alzheimer’s disease frequently display apathy, and some patients may also present with depression, anxiety, irritability, agitation, aggression, hallucinations or delusions.^2,5^ Neuropsychiatric symptoms are especially prevalent in frontotemporal dementia. Five of the six core diagnostic features for the behavioural variant of frontotemporal dementia involve neuropsychiatric, rather than cognitive symptoms.^6^ Behavioural symptoms are also known to occur in the other frontotemporal dementia syndromes—the nonfluent and semantic variants of primary progressive aphasia, and the frontotemporal dementia-spectrum disorders corticobasal syndrome and progressive supranuclear palsy—Richardson syndrome.^7–12^

Clinical diagnostic criteria, such as for behavioural variant frontotemporal dementia, pull together multiple symptoms with a goal of optimizing diagnostic sensitivity and specificity, but this strategy for symptom clustering does not clarify the neuroanatomy that is relevant to specific behaviours or point to a therapeutic approach. Patients with one neurodegenerative syndrome do not all present with the same combination of behavioural symptoms, and patients with different syndromes may display overlapping behavioural features.^13^ For example, similar eating changes and repetitive behaviours may occur both in behavioural variant frontotemporal dementia and semantic variant primary progressive aphasia. Many studies seek to identify the neural correlates of individual behaviours in a neurodegenerative disease. Individual symptoms, such as apathy, may be hard to reproducibly measure, and a behaviour given a particular label in one degenerative disease may differ from the behaviour given the same label in a different one. They might, therefore, have different neural underpinnings that would be missed by studying the correlates of a single behaviour in neurodegenerative diseases. By looking at behavioural profiles (i.e. sets of symptoms that tend to occur together independent of diagnosis), the specificity may be increased, leading to the identification of brain regions that are consistently affected when these symptom clusters occur.

In this study, we employed a 109-item behavioural questionnaire to capture a range of neuropsychiatric symptoms in patients with frontotemporal dementia- and Alzheimer’s disease-related syndromes. Our objective was to identify the shared behavioural profiles in these diseases and to identify their underlying neural correlates.

## Materials and methods

### Participants

We searched the UCSF Memory and Aging Center database for patients who completed the 109-item behavioural questionnaire from 2004 to 2010. Subjects underwent an evaluation as a part of research studies at the Memory and Aging Center. All patients were assessed by an interdisciplinary team of clinicians consisting of neurologists, neuropsychologists, psychiatrists and nurses. Patients underwent extensive behavioural, neuroimaging and neuropsychological assessment, and diagnostic criteria were met at the time of evaluation.^14–19^ The neuropsychological assessment included tests for memory, language, visuospatial ability and executive function, which was previously described.^20^ Overall functional status was assessed using the Clinical Dementia Rating Scale (CDR).^21^ Consent was obtained for all individuals included in the study according to the Declaration of Helsinki and has been approved by the University of California, San Francisco (UCSF) Committee on Human Research. We selected all patients with an Alzheimer’s disease-associated or frontotemporal dementia-spectrum diagnosis. Included participants had a clinical diagnoses of Alzheimer’s-type dementia, behavioural variant frontotemporal dementia, corticobasal syndrome, mild cognitive impairment, nonfluent variant primary progressive aphasia, progressive supranuclear palsy—Richardson syndrome, logopenic variant primary progressive aphasia or semantic variant primary progressive aphasia. Healthy controls were included as they provided an increased range of values, which helps create more meaningful correlations in a principal component analysis (PCA). A total of 326 participants were selected.

### Behavioural questionnaire

The behavioural questionnaire was designed to capture a broad range of symptoms that can be observed in frontotemporal dementia or other neurodegenerative diseases. The symptom list was originally derived from a published 55-item informant-based interview from Manchester University.^22^ After addition, subtraction and modification of questions, the behavioural questionnaire included 109 items to assess behaviour on several domains: Social, Sensory, Eating and Vegetative, Compulsions and Rituals, Environmental Dependency and Hallucinations and Delusions. Each item had six response options describing the frequency of occurrence for each symptom: Never, A few times a month, A few times a week, Daily, Incessant and Had Problem/not anymore. Caregivers were asked to check the box that best describes the patient’s behavioural symptoms within the past 6 months. Healthy controls completed the assessment themselves.

### Statistical analyses

The statistical analyses were conducted in R Project using RStudio.^23,24^ Analysis of variance (ANOVA) and chi-square tests were used, where appropriate, to compare demographics information in Table 1. Two-tailed tests were used with an alpha level of 0.05 to determine significance.

**Table 1:** Participant demographics and characteristics

	Ad	bvFTD	CBS	HC	lvPPA	MCI	nfvPPA	PSP-RS	svPPA	Test statistic	*P*-value
	*n* = 64	*n* = 33	*n* = 18	*n* = 55	*n* = 3	*n* = 63	*n* = 12	*n* = 12	*n* = 21		
Age	65.5 (10.2)	63.2 (6.4)	64.5 (5.3)	65.8 (11.5)	67.4 (12.0)	68.2 (10.2)	66.3 (8.9)	70.0 (7.1)	65.4 (7.0)	*F* = 1.29	0.254
Sex										*χ* ^2^ = 9.20	0.239
F	26 (40.6%)	15 (45.5%)	9 (50%)	30 (54.5%)	3 (100%)	33 (52.4%)	9 (75%)	3 (25%)	9 (42.9%)		
M	38 (69.4%)	18 (54.5%)	9 (50%)	25 (45.5%)	0 (0%)	30 (47.6%)	3 (25%)	9 (75%)	12 (57.1%)		
Race										*χ* ^2^ = 9.41	0.225
White	62 (96.9%)	28 (84.8%)	14 (77.8%)	53 (96.4%)	3 (100%)	59 (93.7%)	10 (83.3%)	10 (83.3%)	20 (95.2%)		
Not White	2 (3.1%)	5 (15.2%)	2 (11.1%)	2 (3.6%)	0 (0%)	3 (4.8%)	1 (8.3%)	2 (16.7%)	1 (4.8%)		
Did not specify	0 (0%)	0 (0%)	2 (11.1%)	0 (0%)	0 (0%)	1 (1.6%)	1 (8.3%)	0 (0%)	0 (0%)		
Education	15.7 (2.9)^e^	16.1 (2.5)	15.3 (2.5)	16.9 (2.1)	16.7 (4.2)	17.2 (2.3)^a^	15.2 (2.3)	17.6 (3.5)	15.5 (2.4)	*F* = 3.48	0.001
CDR	1.0 (0.5)^bdef^	1.5 (0.9)^acdef^	0.7 (0.6)^bd^	0.0 (0.1)^abcegh^	0.7 (0.3)	0.4 (0.2)^abdgh^	0.5 (0.4)^abgh^	1.1 (0.5)^deh^	1.1 (0.6)^def^	*F* = 36.81	<0.001
CDR-SB	5.7 (3.4)^bdef^	8.3 (4.9)^acdef^	4.0 (3.2)^bde^	0.1 (0.3)^abcgh^	2.5 (1.8)	1.1 (1.2)^abcgh^	2.8 (2.0)^abdgh^	7.3 (3.0)^defh^	6.1 (3.8)^def^	*F* = 42.93	<0.001
MMSE	20.8 (6.9)^deh^	22.9 (7.9)^deh^	24.7 (5.7)^dh^	29.6 (0.7)^abch^	21.0 (5.3)	28.6 (1.5)^abh^	23.7 (7.8)^h^	25.8 (3.4)^h^	16.1 (9.7)^abcdef^	*F* = 22.25	<0.001

ANOVA or chi-square tests, when appropriate, were used to compare the groups. lvPPA patients were excluded from statistical analyses for insufficient sample size. Means and (standard deviations) are presented for continuous variables, and counts and (percentages within groups) were used for categorical variables. Bonferroni-corrected *post hoc* pairwise comparisons using *t*-tests were applied to significant ANOVAs: a = significantly different from Ad; b = significantly different from bvFTD; c = significantly different from CBS; d = significantly different from HC; e = significantly different from MCI; f = significantly different from nfvPPA; g = significantly different from PSP-RS; h = significantly different from svPPA. Five participants did not have education data; one participant did not have CDR and CDR-SB data; 22 participants did not have MMSE data. CDR, Clinical Dementia Rating Scale total; CDR-SB, Clinical Dementia Rating Scale—Sum of the Boxes; MMSE, Mini-Mental State Examination; bvFTD, behavioural variant frontotemporal dementia; Ad, Alzheimer’s disease; CBS, corticobasal syndrome; PSP-RS, progressive supranuclear palsy-Richardson syndrome; nfvPPA, nonfluent variant primary progressive aphasia; svPPA, semantic variant primary progressive aphasia; MCI, mild cognitive impairment; lvPPA, logopenic variant primary progressive aphasia.

#### Principal component analysis

In preparation for PCA, the distribution of missing values in the data set was examined. The imputation of missing values is optimal when the data are ‘missing at random’^25^; however, in our data, observations with more than five missing values had the entire sections of the questionnaires missing, making imputation inappropriate. Thus, participants were excluded if they had more than five missing items on the behavioural questionnaire. K-nearest neighbours imputation was used on data with five or less missing items. Two hundred and eighty-nine patients and controls were included in the data set.

Prior to entering the data into a PCA, response options were recoded numerically (1–6) in order of increasing frequency. The option ‘had problem/not anymore’ was coded as two. To determine how many components to use, a scree plot was produced and visually inspected for a clear inflection point suggesting a significant drop-off in variance explained after a certain number of components.

The data were then entered into a PCA using ordinary least squares to find the minimum residual solution. To remove the effect of outliers—at the level of both participant and variable—we used an iterative outlier exclusion process.^26^ For participants, we defined an outlier as an observation with any component score more than six standard deviations away from the mean. For questions, we looked at their communalities (the sums of squared component loadings per variable over all components), which reflect the contribution of each variable to the overall variance explained. Questions with a communality lower than 0.1 were excluded. This iterative process first examined observations for participant-level outliers, excluding them and re-running the solution if they were found. After no outlier observations remained, we then looked for variables with low communalities. The whole process was repeated until no participant-level outliers or low-communality variables remained. Visual inspection of the scree plot revealed an inflection after the seventh eigenvalue, justifying a seven-component solution (Supplementary Fig. 3). The final solution to the iterative component analysis excluded eight observations and six variables, yielding a final data set of 281 observations (participants) and 103 questions. The demographic and descriptive data for this sample are found in Table 1. All demographic and descriptive data were linked within a year (with >75% of data within a week and only two individuals outside of 6 months) to the time of completing the behavioural questionnaire. In an effort to understand the degree to which each component correlated with disease severity, a series of regressions were performed with the Clinical Dementia Rating scale Sum of Boxes (CDR-SB) predicting each component (controlling for age, sex and diagnosis).

### Neuroimaging

#### Acquisition/preprocessing

The first available brain MRI scan within 6 months of the behavioural questionnaire was used for imaging analyses. Forty-seven participants did not have images within the 6-month window. Structural T1 MRI images were acquired on one of three scanners (1.5 T, 3 T or 4 T) at the UCSF Neuroscience Imaging Center. Acquisition parameters for all three scanners have been published previously.^27–29^ All images were visually inspected for motion and artefacts. Statistical Parametric Mapping (SPM) 12 default parameters were used in all preprocessing steps. Images were corrected for bias field, segmented and modulated/warped to Montreal Neurological Institute space. Segmented grey matter images were visually inspected after preprocessing. Eight images were removed for insufficient quality. Grey matter images were smoothed with an 8 mm full width at half-maximum Gaussian kernel. As a final step, diagnostic groups were inspected for sufficient size. There were three logopenic variant primary progressive aphasia patients with imaging data, two of which met the diagnostic criteria for Alzheimer’s disease at the time of testing and were reclassified as Alzheimer’s disease for the voxel-based morphometry (VBM) analyses. The other did not meet criteria for Alzheimer’s disease at the time of evaluation and was excluded in the VBM analyses. Healthy controls were included in all imaging analyses. Two hundred and twenty-five images were included in the final data set.

#### Structural voxel-based morphometry

Whole-brain VBM analysis was performed on grey matter images using SPM12. Multiple regressions (controlling for age, sex, scanner type and total intracranial volume) were performed across all diagnoses on each of the seven components. To account for the effect of atrophy typical of specific degenerative diseases, an additional analysis was performed controlling for diagnoses. To reduce the number of covariates in our model, diagnoses were dummy coded and consolidated into three broad groups—those commonly associated with underlying frontotemporal lobar degeneration (semantic variant primary progressive aphasia, behavioural variant frontotemporal dementia, nonfluent variant primary progressive aphasia and progressive supranuclear palsy—Richardson syndrome), those associated with underlying Alzheimer’s disease and those with less predictable clinicopathological association (corticobasal syndrome). Patients with mild cognitive impairment were evaluated with longitudinal diagnostic data, if available, to determine a progression to Alzheimer’s disease. Only mild cognitive impairment patients who met diagnostic criteria for Alzheimer’s disease at a later time point were coded in the Alzheimer’s disease group (*n* = 14). Two mild cognitive impairment patients met diagnostic criteria at a later time point for frontotemporal dementia and were coded with the frontotemporal lobar degeneration group. Patients that did not progress to frontotemporal dementia or Alzheimer’s disease were coded with the healthy controls (*n* = 36). The threshold for statistical significance was set at peak-level *P* < 0.05 after family-wise error (FWE) correction for multiple comparisons. Results were examined using BSPMVIEW^30^, a MATLAB extension at both peak-level FWE *P* < 0.05 and at a level of *P* < 0.001 uncorrected for multiple comparisons with a minimum extent threshold of 10. Voxel-based morphometry maps were visualized and produced using MRIcroGL 64.^31^ To confirm the presence of the expected diagnosis-specific atrophy patterns in this cohort, a VBM analysis was performed to compare each diagnostic group with healthy controls (Supplementary Fig. 2).

### Data availability

Data generated by the UCSF Memory and Aging Center are available upon request. All data requests can be submitted through the UCSF Memory and Aging Center Resource Request Form (http://memory.ucsf.edu/resources/data). Academic, not-for-profit investigators with Institutional Review Board approval from the UCSF Human Research Protection Program can request data for research studies. The UCSF Human Research Protection Program will not review the application until the UCSF Memory and Aging Center Executive Committee has signed off on the proposal and consent form. Data are not publicly available because they contain information that could compromise the privacy of the participants.

## Results

The diagnostic groups did not differ in age, sex and race/ethnicity (see Table 1). While the participants were largely evenly distributed in sex, all groups were predominantly white, older individuals. Years of formal education differed across groups; however, Bonferroni corrected *post hoc* tests revealed significant pairwise comparisons between just the Alzheimer’s disease and mild cognitive impairment groups. Unsurprisingly, the groups differed in measures of dementia severity (CDR) and global cognitive performance [Mini-Mental State Examination (MMSE)].

**Figure 1:**
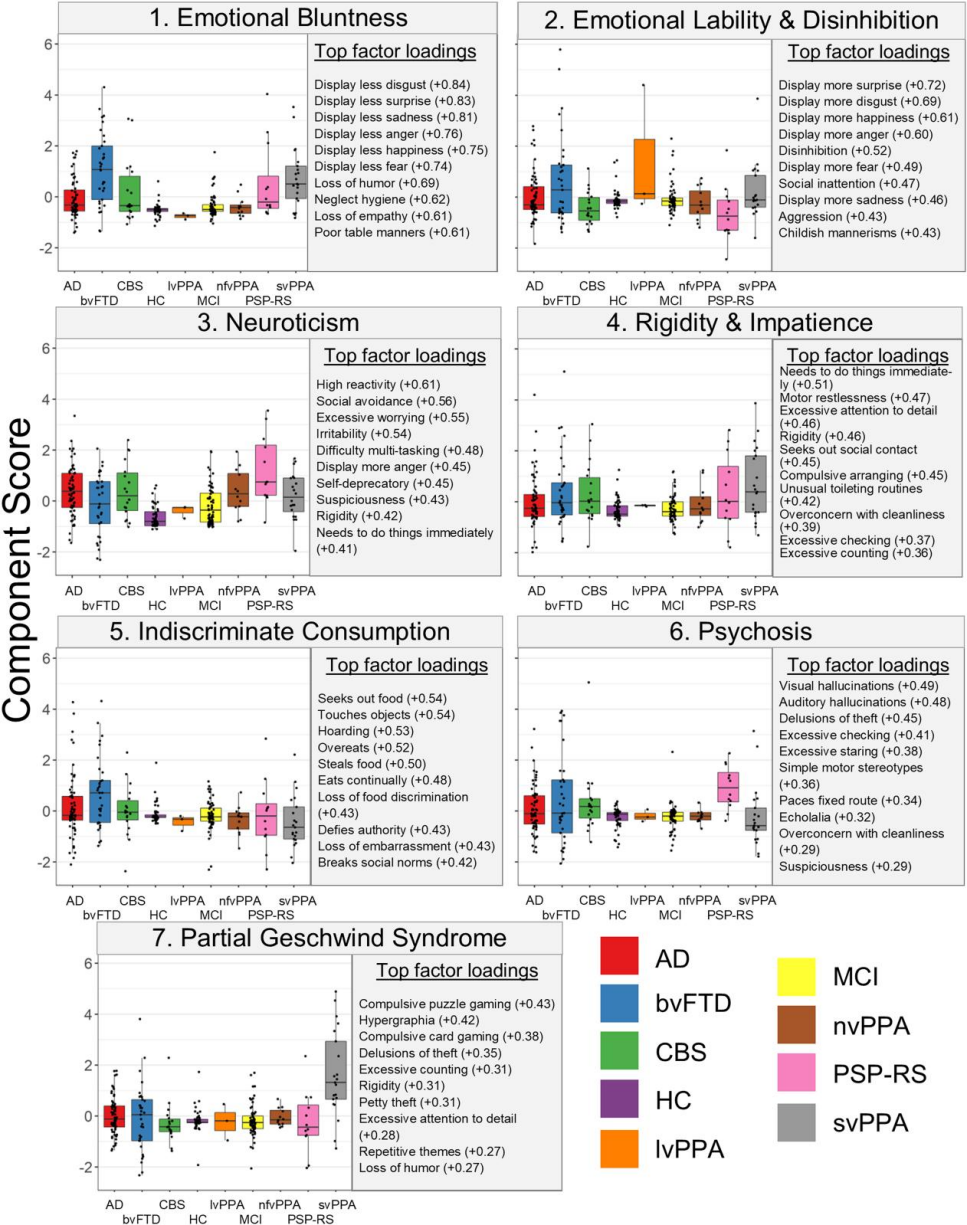
**Box plots for each of the component scores across diagnosis.** The top 10 strongest loadings for each component are presented to the right of the box plots across diagnosis. Box plots visually represent the distribution of scores for each competent across diagnostic groups. Statistical analyses were not performed. bvFTD, behavioural variant frontotemporal dementia; Ad, Alzheimer’s disease; CBS, corticobasal syndrome; PSP-RS, progressive supranuclear palsy-Richardson syndrome; nfvPPA, nonfluent variant primary progressive aphasia; svPPA, semantic variant primary progressive aphasia; MCI, mild cognitive impairment; lvPPA, logopenic variant primary progressive aphasia; HC, healthy controls.

The PCA results were examined, and behavioural patterns in the top loading variables for each component were identified. Figure 1 includes the top 10 loadings for each component, as well as box plots that show the distribution of scores across groups. For full component loadings, see Supplementary Table 2. Box plots examining the distribution of scores across the combined diagnostic groups used in the follow-up diagnosis controlled VBM analyses are presented in Supplementary Fig. 4. The Kaiser–Meyer–Olkin index was measured by using the psych package in R and was found to be 0.87, well above the sampling adequacy typically considered to be acceptable.^32^ The proportion of variance explained by all seven components was 44%. Regressions with CDR-SB on each component revealed that CDR-SB significantly predicted only Component 1, *b* = 1.18, *t*(268) = 6.25, *P* = <0.001, and Component 5, *b* = 0.80, *t*(268) = 4.63, *P* = <0.001, after Bonferroni corrections for multiple comparisons.

**Figure 2:**
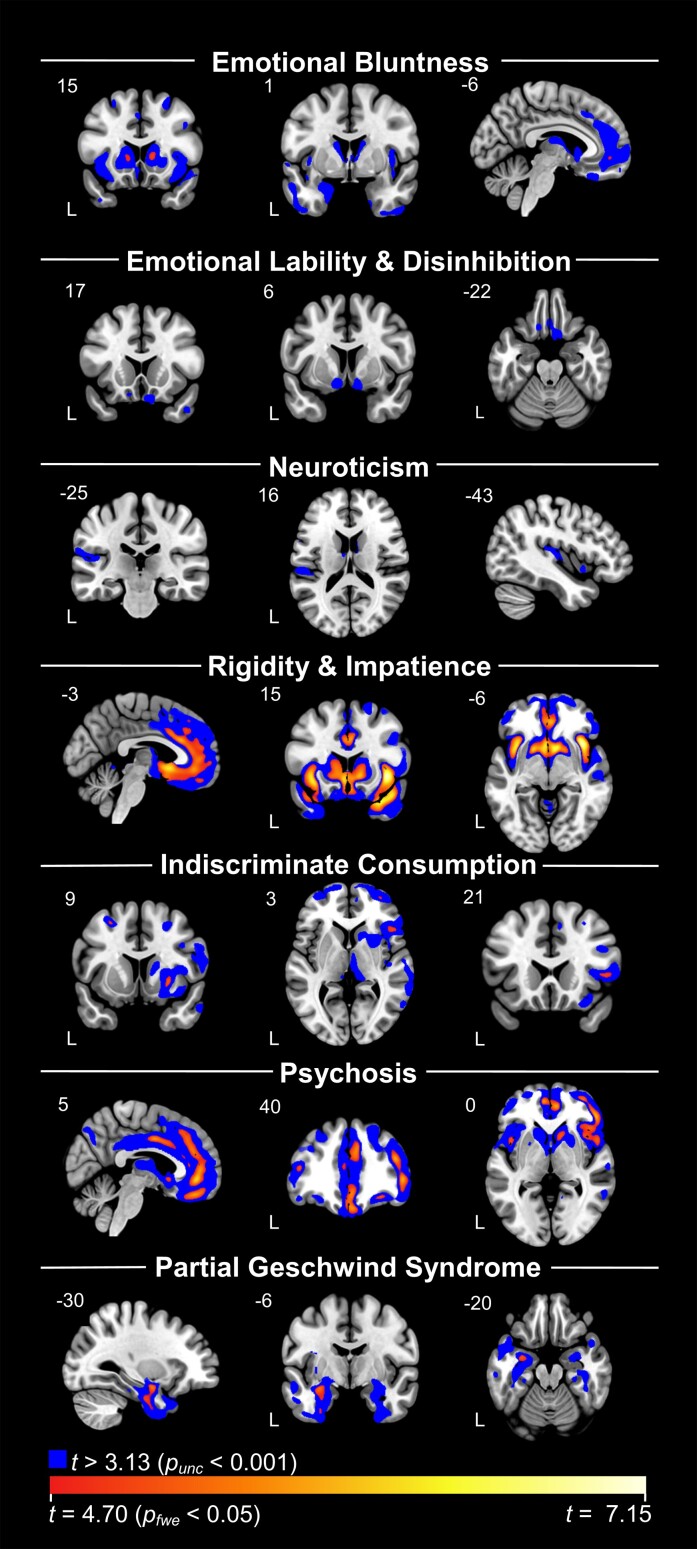
**VBM analyses with each component across diagnosis**. Blue represents results at uncorrected *P* < 0.001 levels. Red/yellow/white spectrum represents findings that survived FWE corrections at *P* < 0.05. Components 2 and 3 did not have any findings at corrected levels. Each analysis controlled for total intracranial volume, age, sex and scanner type (dummy coded).

FWE-corrected significant clusters from the VBM analysis on each of the seven components across diagnoses are presented in Table 2. Maps that include FWE-corrected clusters overlayed on uncorrected *P* < 0.001 clusters are shown on Fig. 2. The results of VBM analyses controlling for diagnosis are presented in Supplementary Table 1 and Supplementary Fig. 1. Results from the VBM analyses comparing each dementia group with healthy controls are presented in Supplementary Fig. 2. Areas of volume loss in the dementia groups when compared to healthy controls were consistent with expected patterns of atrophy of their disease. Patients with mild cognitive impairment did not show significant volume loss compared to healthy controls at FWE-corrected *P* < 0.05 or at uncorrected *P* < 0.001 thresholds, substantiating their grouping with healthy controls when controlling for diagnosis.

**Table 2:** Anatomical correlates of component scores

Component	Anatomical region	Cluster extent (mm^3^)	Peak MNI coordinates			Max *T*
			*x*	*y*	*z*	
Comp. 1						
	Frontal middle 2, R	60	41	36	33	5.18
	Frontal middle 2, R	22	33	47	26	5.06
	Caudate, R	84	12	15	5	5.03
	Caudate, L	40	−12	15	3	4.88
	Frontal medial orbital, L	11	−6	48	−6	4.82
Comp. 2						
						
Comp. 3						
						
Comp. 4						
	Insula, R	4749	38	17	−5	7.15
	Temporal pole superior, R	4749	32	15	−29	6.52
	Temporal middle, R	4749	59	−2	−21	6.03
	Caudate, R	9402	8	11	−8	6.63
	Insula, L	9402	−38	11	−3	6.44
	Putamen, L	9402	−17	11	−6	6.07
	Rectus, L	112	−2	48	−21	5.38
	Frontal superior 2, R	26	29	57	0	5.19
	Frontal superior medial, R	103	5	29	45	5.17
	Frontal middle 2, R	43	35	8	59	5.17
	Frontal superior medial, R	21	11	48	42	5.08
	Frontal superior 2, R	21	23	57	21	5.06
	Temporal pole middle, R	25	44	11	−42	5.04
	Frontal superior 2, R	29	23	51	33	4.92
	Frontal superior medial, R	14	5	51	29	4.91
Comp. 5						
	Putamen, R	63	27	9	−3	5.28
	Frontal superior 2, R	87	29	56	11	5.23
	Frontal middle 2, L	17	−27	9	53	5.11
	Frontal inferior triangularis, R	52	48	21	5	5.08
	Frontal middle 2, R	14	42	51	−9	4.88
	Frontal middle 2, R	18	41	53	18	4.87
Comp. 6						
	Frontal medial orbital, R	2500	5	45	−6	6.12
	Cingulate middle, R	2500	8	36	35	5.84
	Cingulate anterior, L	2500	−5	41	14	4.82
	Frontal middle 2, R	2326	45	45	5	6.07
	Frontal inferior operculum, R	2326	54	18	12	5.94
	Insula, R	2326	35	15	6	5.79
	Cingulate middle, R	464	5	0	38	5.72
	Frontal middle 2, R	39	39	26	42	5.50
	Frontal inferior operculum, R	79	47	18	30	5.44
	Insula, L	137	−42	12	3	5.37
	OFC anterior, R	171	29	38	−14	5.32
	Caudate, R	336	11	14	−3	5.26
	Frontal inferior triangularis, L	36	−45	39	12	5.26
	Cingulate middle, L	37	−6	20	38	4.90
	Cingulate anterior, L	12	−5	38	−3	4.89
	Frontal superior 2, R	11	26	6	60	4.88
	Cingulate anterior, L	14	−8	48	17	4.84
Comp. 7						
	Amygdala, L	294	−30	−5	−17	5.40
	Fusiform, L	294	−38	−14	−35	5.02

All clusters presented are *P*_FWE_ < 0.05. Minimum extent = 10. Table shows all local maxima separated by more than 20 mm. Regions were labelled using the AAL2 atlas. *x*, *y* and *z* = Montreal Neurological Institute (MNI) coordinates in the left–right, anterior–posterior and inferior–superior dimensions, respectively. All contrasts are negative (atrophy related to higher component scores). No clusters survived for positive contrasts. OFC, orbitofrontal cortex.

### Component 1

#### Principal component analysis

Component 1 was predominantly characterized by behaviours related to reduced emotional display, as well as loss of embarrassment and empathy. Visual inspection of the box plots revealed that across diagnoses, behavioural variant frontotemporal dementia and semantic variant primary progressive aphasia patients displayed the highest scores on this component (Fig. 1, statistical comparisons between diagnostic groups are included in Supplementary Table 3).

#### Imaging

Inspection of uncorrected maps (*P* < 0.001) indicated that high Component 1 scores were associated with low volume in the bilateral striatum, insula, medial frontal areas including pregenual anterior cingulate cortex and anterior temporal regions, including amygdala. After correction for multiple comparisons (*P*_FWE_ < 0.05), significant regions included the bilateral caudate, right middle frontal gyrus and left ventromedial prefrontal cortex (Table 2). We found a similar, though less extensive pattern after controlling for diagnosis, with key areas persisting at *P*_unc_ < 0.001, including right caudate, right middle frontal gyrus and medial frontal areas.

### Component 2

#### Principal component analysis

In contrast to the emotional blunting of Component 1, Component 2 was driven by heightened emotional display and disinhibition. Patients with behavioural variant frontotemporal dementia, semantic variant primary progressive aphasia and logopenic variant primary progressive aphasia (although with a small sample size) displayed somewhat higher scores on this component.

#### Imaging

No clusters survived FWE correction. At uncorrected thresholds (*P* < 0.001), volume loss in the bilateral posterior and medial orbitofrontal cortex and ventral aspects of the striatum (including nucleus accumbens) predicted higher scores on this component. When controlling for diagnosis, the findings generally remained unchanged.

### Component 3

#### Principal component analysis

Increased emotional reactivity characterized the behaviours primarily associated with this component, with particular emphasis on facets related to neuroticism (e.g. social avoidance, excessive worry and anger).^33^ On average, patients with progressive supranuclear palsy—Richardson syndrome—had the highest scores on this component, followed by those with Alzheimer’s disease, whereas controls scored lowest.

#### Imaging

While no clusters survived FWE correction, at uncorrected levels (*P* < 0.001), higher scores correlated with reduced volume in the left mid-to-posterior insula and left parietal operculum, as well as the bilateral thalamus and caudate. Additionally, there were smaller clusters in the left middle frontal gyrus and the left posterior middle temporal gyrus. No clusters remained significant at this threshold when controlling for diagnosis.

### Component 4

#### Principal component analysis

Component 4 was driven by behaviours related to restlessness (including impatience and the need to do things immediately) and compulsion (excessive attention to detail, checking, counting, cleaning, arranging and rigidity). Patients with semantic variant primary progressive aphasia displayed slightly higher scores, though a range of component scores were seen in patients across many diagnostic groups.

#### Imaging

Higher scores on Component 4 were correlated (*P*_FWE_ < 0.05) with atrophy in large clusters that involved the bilateral insula, basal ganglia and anterior temporal lobes, as well as the anterior cingulate cortex and medial frontal areas (Table 2). While this set of regions resembled the pattern of atrophy associated with behavioural variant frontotemporal dementia, findings largely persisted in a similar pattern after controlling for diagnosis.

### Component 5

#### Principal component analysis

Component 5 was primarily characterized by behaviours related to indiscriminate consumption of food. Patients with behavioural variant frontotemporal dementia had the highest scores on this component and semantic variant primary progressive aphasia the lowest; however, there was variance on this component across all neurodegenerative diagnoses, with the exception of the few logopenic variant primary progressive aphasia patients.

#### Imaging

Negative correlations showed predominantly right-sided atrophy in superior and middle fontal regions, basal ganglia (predominantly the putamen) and frontal operculum at *P*_FWE_ < 0.05. At uncorrected levels (*P* < 0.001), clusters remained asymmetric, including large clusters in the frontal lobes, basal ganglia, thalamus and dorsal and ventral insula. Controlling for diagnosis had little effect on these results.

### Component 6

#### Principal component analysis

Psychotic symptoms, both hallucinations and delusions, as well as some repetitive behaviours, were the symptoms with the highest loadings for this component. Progressive supranuclear palsy—Richardson syndrome—patients were notably high relative to the other diagnostic groups, with a wide range of data among patients with Alzheimer’s disease and behavioural variant frontotemporal dementia, and low scores among patients with semantic variant primary progressive aphasia.

#### Imaging

While uncorrected maps indicate an association between high component scores and bilateral frontal and subcortical atrophy, FWE correction revealed that the strongest effects were observed in mostly right-sided regions, including a large cluster that spanned from the medial orbitofrontal cortex to the middle cingulate, another that included the middle frontal gyrus, inferior frontal operculum and insula and a cluster in the right caudate. Controlling for diagnosis reduced cluster sizes; however, the pattern of findings remained consistent and key clusters remained significant.

### Component 7

#### Principal component analysis

The top loadings for Component 7 included hypergraphia and certain complex compulsive interests, such as repetitively playing puzzle and card games. Patients with semantic variant primary progressive aphasia had notably higher scores on this component.

#### Imaging

Volume loss in the anterior temporal lobes was associated with higher scores on this component. At *P*_unc_ < 0.001, this included medial more than lateral, left greater than right anterior temporal regions. At *P*_FWE_ < 0.05, significant regions involved the left amygdala and the left fusiform. When controlling for diagnosis, anterior temporal lobe clusters remained, with left amygdala remaining significant after FWE correction.

## Discussion

While behavioural symptoms are a well-known feature of many neurodegenerative diseases, there is limited understanding of how these symptoms interrelate within and across diagnoses. In a large cohort of patients with frontotemporal dementia- and Alzheimer’s disease-related syndromes, we identified components from a 109-item symptom questionnaire that captured seven behavioural phenotypes: emotional bluntness, emotional lability and disinhibition, neuroticism, rigidity and compulsion, indiscriminate consumption, psychosis and elements reminiscent of the Geschwind syndrome. Each of these components related to a pattern of volume loss that helps inform the structural underpinnings of each symptom cluster.

### Component 1: emotional bluntness

Widespread emotional blunting was associated with low volume in the striatum, insula, medial frontal and anterior temporal regions bilaterally. These areas have been linked to reduced emotional reactivity in dementia patients. For example, atrophy in the left anterior temporal regions and left anterior insula has been associated with diminished autonomic reactivity to emotional stimuli in both semantic variant primary progressive aphasia and nonfluent variant primary progressive aphasia patients.^34^ Insula atrophy has been repeatedly associated with emotional changes including diminished facial reactivity,^35^ self-reported experience and physiological responses to emotional stimuli.^36–38^ Furthermore, reduced volume in the amygdala, a region that is well established as being involved in emotion generation, has been associated with blunting of autonomic response to affective stimuli^35^ and self-reported emotional experience in dementia.^37^ The integrity of the left caudate, another key region associated with this component, is linked to diminished skin conductance response to negative stimuli in behavioural variant frontotemporal dementia and semantic variant primary progressive aphasia.^35^ In addition to emotional blunting, behaviours such as lack of self-consciousness and loss of empathy were loaded heavily on this component, which is consistent with evidence that reduced pregenual anterior cingulate cortex volume is a significant predictor of impaired self-conscious emotional reactivity,^39^ and that cingulate cortex, insula and amygdala are key areas involved in embarrassment and guilt.^40^ Taken together, these findings suggest that volume loss in distributed regions of the salience network that are involved in both interoceptive processing and emotion generation may lead to disruptions in affective processes that contribute to an overall blunting of emotions. While patients with behavioural variant frontotemporal dementia commonly exhibit these behaviours and scored highest on this component, the persistence of small clusters after controlling for diagnosis suggests that these regions influence emotional symptoms across diseases.

### Component 2: emotional lability and disinhibition

Greater disinhibition is linked to atrophy of regions identified on uncorrected maps, including orbitofrontal/ventromedial prefrontal cortex and ventral striatum.^41–43^ Furthermore, these regions are key areas involved in emotion regulation,^44^ and atrophy in these regions is associated with heightened positive emotional reactivity in patients with frontotemporal dementia.^45^ Additionally, there was a cluster in the right anterior temporal lobe, a region that has also been linked to disinhibition.^46^ Our findings suggest that these regions influence emotional reactivity and regulation, contributing to disinhibition across diagnoses since these clusters remained relatively unchanged when controlling for diagnosis.

### Component 3: neuroticism

Behaviours that loaded heavily on Component 3 included several symptoms related to enhanced response to negative affect or neuroticism (irritability, anxiety and self-consciousness). The neural correlates of aspects of neuroticism have been studied through varied experimental instruments. While numerous brain structures have been implicated, a meta-analysis found a negative correlation between neuroticism and volume in regions that partially overlap with the findings from our study at the 0.001 uncorrected threshold, including left thalamus, left middle temporal gyrus and left caudate.^47^ Additionally, in preclinical Huntington’s disease patients, increased irritability was associated with striatal atrophy.^48^ The clusters with the largest effect sizes in this study linked high Component 3 scores with atrophy of posterior insula and parietal operculum. Further study can help explore the behavioural effect of atrophy in these regions, which are known to be associated with somatosensory processing.

### Component 4: rigidity and impatience

Fixed habits and repetitive or compulsive behaviours are implicated in numerous neurological and psychiatric conditions, but may have heterogeneous manifestations, from simple stereotypies to anxiety-driven obsessions, to complex compulsions. This component grouped several complex compulsions, such as counting and arranging, with rigidity and impatience. This cluster of symptoms demonstrated variability of scores across diagnoses and linked symptom severity to subcortical, medial frontal, insula and temporal atrophy. An association between compulsions and basal ganglia atrophy, as well as temporal lobe atrophy in frontotemporal dementia, has previously been identified.^49,50^ In addition, past work has shown that obsessive–compulsive disorder patients displayed reduced activity in medial frontal areas, cingulate and caudate compared to healthy controls in a task switching paradigm, demonstrating greater cognitive inflexibility (i.e. rigidity), suggesting that these areas are involved in repetitive behaviours across diagnoses.^51^ Consistent with previous work, these findings suggest that a disconnect between goal-directed and habitual actions may contribute to these behaviours and be mediated by the basal ganglia and other regions associated with this cluster.^52^

### Component 5: indiscriminate consumption

As overeating and craving of sweet foods form a core diagnostic feature of behavioural variant frontotemporal dementia, it is unsurprising that patients with behavioural variant frontotemporal dementia had the strongest loadings among the diagnostic groups on Component 5, which brought together multiple features of indiscriminate consumption. The negative correlation between high Component 5 scores with volume in right insula, striatum and frontal atrophy is consistent with past research that has linked overeating and increased reward-seeking behaviour to atrophy in these structures in frontotemporal dementia patients.^53–55^ Other behaviours that relate to reward seeking had more moderate loadings on this component (increased alcohol consumption—0.22, hypersexuality—0.21). A case study of frontotemporal dementia patients with Diogenes syndrome suggests that hoarding behaviours also involve similar right, frontolimbic–striatal systems.^56^ Controlling for diagnosis had little effect on these clusters, suggesting that these brain–behaviour correlations are not specific to behavioural variant frontotemporal dementia.

### Component 6: psychosis

Hallucinations and delusions occur in a wide range of neurodegenerative diseases, including Alzheimer’s disease and frontotemporal lobar degeneration.^57,58^ Their neural correlates have been studied in both patients with neurodegenerative disease and psychiatric illness,^59,60^ with findings that implicate numerous brain regions including the sensory cortices, subcortical regions, cingulate and insula to prefrontal connections. While localization of psychotic symptoms has varied across studies in sporadic and genetic frontotemporal dementia,^61,62^ the right-predominant atrophy associated with this component is consistent with a prior report of asymmetric atrophy in patients with frontotemporal dementia and psychotic symptoms.^63^ Furthermore, the insula and the anterior cingulate cortex have been more consistently associated with psychotic behaviours. These regions are central nodes in the salience network that aid in multisensory integrative process. It has been hypothesized that disruption to normal salience network activity (e.g. attenuated coactivation of the insula and cingulate cortex) results in a failure to correctly process internally generated mental activity leading to an exacerbation of psychotic symptoms.^64^ This complex process likely involves dysfunction at multiple levels, including aberrant processing of sensory symptoms and error monitoring, which may contribute to the complexity and heterogeneity in imaging findings. The fact that imaging correlates for this component were robust when controlling for diagnosis suggests that this component captures a shared symptom and anatomic profile across multiple psychotic symptoms and degenerative diagnoses.

### Component 7: partial Geschwind syndrome

The Geschwind syndrome includes hypergraphia, hyperreligiosity, atypical sexuality, intensified mental life and circumstantiality and has been described in some patients with temporal lobe epilepsy.^65^ Component 7 includes strong loadings for hypergraphia and several symptoms that are consistent with the type of solitary pursuits that have been associated with intensified mental life (compulsive puzzles, playing of card games such as solitaire and excessive attention to detail). Dogmatism, which in some cases is related to hyperreligiosity, was loaded moderately on this component (0.24), though hyposexuality did not have a strong loading. While this component did not recreate the full Geschwind syndrome, the temporal-predominant imaging correlates are consistent with other prior studies that have implicated the medial and lateral portions of the temporal lobe with hypergraphia and compulsions,^49,66–70^ with varying interpretations of the underlying mechanism for hypergraphia, including changes in emotional reactivity,^71^ or in a prior case report of semantic variant primary progressive aphasia, a possible functional facilitation of verbal creativity.^72^

### Limitations

Limitations of the current study include reliance on self or informant report for the presence and frequency of symptoms. Although the intent of this study is not to validate this measure as a diagnostic or prognostic marker of disease, future work could still benefit from a more objective assessment of clinical behaviours. In spite of efforts to account for the effect of diagnosis, disease-specific atrophy patterns may influence the imaging correlates of components that are more strongly associated with specific syndromes. The correlates of certain symptoms may differ across diagnoses, with similar symptoms resulting from lesions to different portions of a common circuit. Future studies could also explore functional changes that may interact with structural ones in driving behaviour.

## Conclusion

Localized neurodegeneration can lead to the development of behavioural symptom clusters across various dementia syndromes. Across a large sample of degenerative disease patients, we have identified a range of behaviours that co-occur using a principal component analysis. Correlations with these symptom profiles and brain volume identified distinct neuroanatomical patterns associated with each component. Largely, these findings persisted when controlling for diagnosis, suggesting that these brain-to-behavioural correlations are informative across multiple degenerative syndromes.

## Supplementary Material

fcad038_Supplementary_Data

## Abbreviations

CDR =: Clinical Dementia Rating Scale
CDR-SB =: Clinical Dementia Rating Scale Sum of Boxes
FWE =: family-wise error
MMSE =: Mini-Mental State Examination
PCA =: principal component analysis
SPM =: statistical parametric mapping
UCSF =: University of California, San Francisco
VBM =: voxel-based morphometry

## References

[1] Assal F , Cummings JL. Neuropsychiatric symptoms in the dementias. Curr Opin Neurol. 2002;15:445–450. 12151841 10.1097/00019052-200208000-00007

[2] Lyketsos CG , Lopez O, Jones B, Fitzpatrick AL, Breitner J, DeKosky S. Prevalence of neuropsychiatric symptoms in dementia and mild cognitive impairment. Results from the cardiovascular health study. JAMA. 2002;288:1475–1483. 12243634 10.1001/jama.288.12.1475

[3] Srikanth S , Nagaraja AV, Ratnavalli E. Neuropsychiatric symptoms in dementia-frequency, relationship to dementia severity and comparison in Alzheimer’s disease, vascular dementia and frontotemporal dementia. J Neurol Sci. 2005;236:43–48. 15964021 10.1016/j.jns.2005.04.014

[4] Ballard C , Day S, Sharp S, Wing G, Sorensen S. Neuropsychiatric symptoms in dementia: Importance and treatment considerations. Int Rev Psychiatry. 2008;20:396–404. 18925489 10.1080/09540260802099968

[5] Lyketsos CG , Carrillo MC, Ryan JM, et al Neuropsychiatric symptoms in Alzheimer’s disease. Alzheimers Dement. 2011;7:532–539. 21889116 10.1016/j.jalz.2011.05.2410 PMC3299979

[6] Rascovsky K , Hodges JR, Knopman D, et al Sensitivity of revised diagnostic criteria for the behavioural variant of frontotemporal dementia. Brain. 2011;134:2456–2477. 21810890 10.1093/brain/awr179 PMC3170532

[7] Thompson SA , Patterson K, Hodges JR. Left/right asymmetry of atrophy in semantic dementia. Neurology. 2003;61:1196–1203. 14610120 10.1212/01.wnl.0000091868.28557.b8

[8] Liu W , Miller BL, Kramer JH, et al Behavioral disorders in the frontal and temporal variants of frontotemporal dementia. Neurology. 2004;62:742–748. 15007124 10.1212/01.wnl.0000113729.77161.c9 PMC2367136

[9] Rosen HJ , Allison SC, Ogar JM, et al Behavioral features in semantic dementia vs other forms of progressive aphasias. Neurology. 2006;67:1752–1756. 17130406 10.1212/01.wnl.0000247630.29222.34

[10] Banks SJ , Weintraub S. Neuropsychiatric symptoms in behavioral variant frontotemporal dementia and primary progressive aphasia. J Geriatr Psychiatry Neurol. 2008;21:133–141. 18474722 10.1177/0891988708316856 PMC2892801

[11] Bruns MB , Josephs KA. Neuropsychiatry of corticobasal degeneration and progressive supranuclear palsy. Int Rev Psychiatry. 2013;25:197–209. 23611349 10.3109/09540261.2013.766154

[12] Harris JM , Jones M, Gall C, et al Co-occurrence of language and behavioural change in frontotemporal lobar degeneration. Dement Geriatr Cogn Disord Extra. 2016;6:205–213. 10.1159/000444848 PMC4913762 27350781

[13] Murley AG , Coyle-Gilchrist I, Rouse MA, et al Redefining the multidimensional clinical phenotypes of frontotemporal lobar degeneration syndromes. Brain. 2020;143:1555–1571. 32438414 10.1093/brain/awaa097 PMC7241953

[14] McKhann G , Drachman D, Folstein M, Katzman R, Price D, Stadlan EM. Clinical diagnosis of Alzheimer’s disease. Neurology. 1984;34:939. 6610841 10.1212/wnl.34.7.939

[15] Litvan I , Agid Y, Calne D, et al Clinical research criteria for the diagnosis of progressive supranuclear palsy (Steele-Richardson-Olszewski syndrome). Neurology. 1996;47:1–9. 8710059 10.1212/wnl.47.1.1

[16] Neary D , Snowden JS, Gustafson L, et al Frontotemporal lobar degeneration. Neurology. 1998;51:1546–1554. 9855500 10.1212/wnl.51.6.1546

[17] Boxer AL , Geschwind MD, Belfor N, et al Patterns of brain atrophy that differentiate corticobasal degeneration syndrome from progressive supranuclear palsy. Arch Neurol. 2006;63:81–86. 16401739 10.1001/archneur.63.1.81

[18] Gorno-Tempini ML , Hillis AE, Weintraub S, et al Classification of primary progressive aphasia and its variants. Neurology. 2011;76:1006–1014. 21325651 10.1212/WNL.0b013e31821103e6 PMC3059138

[19] Rascovsky K , Grossman M. Clinical diagnostic criteria and classification controversies in frontotemporal lobar degeneration. Int Rev Psychiatry. 2013;25:145–158. 23611345 10.3109/09540261.2013.763341 PMC3906583

[20] Kramer JH , Jurik J, Sha SJ, et al Distinctive neuropsychological patterns in frontotemporal dementia, semantic dementia, and Alzheimer disease. Cogn Behav Neurol. 2003;16:211–218. 14665820 10.1097/00146965-200312000-00002

[21] Morris JC . The Clinical Dementia Rating (CDR). Neurology. 1993;43:2412–2414. 10.1212/wnl.43.11.2412-a 8232972

[22] Snowden JS , Bathgate D, Varma A, Blackshaw A, Gibbons ZC, Neary D. Distinct behavioural profiles in frontotemporal dementia and semantic dementia. J Neurol Neurosurg Amp Psychiatry. 2001;70:323–332.. 10.1136/jnnp.70.3.323 PMC1737271 11181853

[23] R Core Team . R: A language and environment for statistical computing. R Foundation for Statistical Computing; 2021.

[24] RStudio Team . RStudio: Integrated development for R. RStudio. Posit Software, PBC; 2020.

[25] Donders ART , van der Heijden GJMG, Stijnen T, Moons KGM. Review: A gentle introduction to imputation of missing values. J Clin Epidemiol. 2006;59:1087–1091. 16980149 10.1016/j.jclinepi.2006.01.014

[26] Linting M , van der Kooij A. Nonlinear principal components analysis with CATPCA: A tutorial. J Pers Assess. 2012;94:12–25. 22176263 10.1080/00223891.2011.627965

[27] Rosen HJ , Gorno–Tempini ML, Goldman WP, et al Patterns of brain atrophy in frontotemporal dementia and semantic dementia. Neurology. 2002;58:198–208. 11805245 10.1212/wnl.58.2.198

[28] Mueller SG , Laxer KD, Barakos J, Cheong I, Garcia P, Weiner MW. Subfield atrophy pattern in temporal lobe epilepsy with and without mesial sclerosis detected by high-resolution MRI at 4 Tesla: Preliminary results. Epilepsia. 2009;50:1474–1483. 19400880 10.1111/j.1528-1167.2009.02010.x PMC2804395

[29] Bettcher BM , Wilheim R, Rigby T, et al C-reactive protein is related to memory and medial temporal brain volume in older adults. Brain Behav Immun. 2012;26:103–108. 21843630 10.1016/j.bbi.2011.07.240 PMC3221922

[30] Spunt B . spunt/bspmview: BSPMVIEW. Published online November 22, 2016. 10.5281/zenodo.168074.

[31] Rorden C , Brett M. Stereotaxic display of brain lesions. Behav Neurol. 2000;12:191–200. 11568431 10.1155/2000/421719

[32] Kaiser HF . An index of factorial simplicity. Psychometrika. 1974;39:31–36.

[33] Tackett JL , Lahey BB. Neuroticism, eds. The Oxford handbook of the five factor model. Oxford library of psychology. Oxford University Press; 2017:39–56.

[34] Hua AY , Chen KH, Brown CL, et al Physiological, behavioral and subjective sadness reactivity in frontotemporal dementia subtypes. Soc Cogn Affect Neurosci. 2019;14:1453–1465. 31993653 10.1093/scan/nsaa007 PMC7137727

[35] Kumfor F , Hazelton JL, Rushby JA, Hodges JR, Piguet O. Facial expressiveness and physiological arousal in frontotemporal dementia: Phenotypic clinical profiles and neural correlates. Cogn Affect Behav Neurosci. 2019;19:197–210. 30488224 10.3758/s13415-018-00658-z

[36] Hoefer M , Allison SC, Schauer GF, et al Fear conditioning in frontotemporal lobar degeneration and Alzheimer’s disease. Brain. 2008;131:1646–1657. 18492729 10.1093/brain/awn082 PMC2544622

[37] Verstaen A , Eckart JA, Muhtadie L, et al Insular atrophy and diminished disgust reactivity. Emotion. 2016;16:903–912. 27148847 10.1037/emo0000195 PMC5009015

[38] Marshall CR , Hardy CJD, Allen M, et al Cardiac responses to viewing facial emotion differentiate frontotemporal dementias. Ann Clin Transl Neurol. 2018;5:687–696. 29928652 10.1002/acn3.563 PMC5989744

[39] Sturm VE , Sollberger M, Seeley WW, et al Role of right pregenual anterior cingulate cortex in self-conscious emotional reactivity. Soc Cogn Affect Neurosci. 2013;8:468–474. 22345371 10.1093/scan/nss023 PMC3624960

[40] Bastin C , Harrison BJ, Davey CG, Moll J, Whittle S. Feelings of shame, embarrassment and guilt and their neural correlates: A systematic review. Neurosci Biobehav Rev. 2016;71:455–471. 27687818 10.1016/j.neubiorev.2016.09.019

[41] Crews FT , Boettiger CA. Impulsivity, frontal lobes and risk for addiction. Pharmacol Biochem Behav. 2009;93:237–247. 19410598 10.1016/j.pbb.2009.04.018 PMC2730661

[42] Osborne-Crowley K , McDonald S. A review of social disinhibition after traumatic brain injury. J Neuropsychol. 2018;12:176–199. 27696753 10.1111/jnp.12113

[43] Zamboni G , Huey ED, Krueger F, Nichelli PF, Grafman J. Apathy and disinhibition in frontotemporal dementia. Neurology. 2008;71:736–742. 18765649 10.1212/01.wnl.0000324920.96835.95 PMC2676948

[44] Etkin A , Büchel C, Gross JJ. The neural bases of emotion regulation. Nat Rev Neurosci. 2015;16:693–700. 26481098 10.1038/nrn4044

[45] Sturm VE , Yokoyama JS, Eckart JA, et al Damage to left frontal regulatory circuits produces greater positive emotional reactivity in frontotemporal dementia. Cortex. 2015;64:55–67. 25461707 10.1016/j.cortex.2014.10.002 PMC4346386

[46] Hornberger M , Geng J, Hodges JR. Convergent grey and white matter evidence of orbitofrontal cortex changes related to disinhibition in behavioural variant frontotemporal dementia. Brain. 2011;134:2502–2512. 21785117 10.1093/brain/awr173

[47] Servaas MN , van der Velde J, Costafreda SG, et al Neuroticism and the brain: A quantitative meta-analysis of neuroimaging studies investigating emotion processing. Neurosci Biobehav Rev. 2013;37:1518–1529. 23685122 10.1016/j.neubiorev.2013.05.005

[48] Van den Stock J , De Winter FL, Ahmad R, et al Functional brain changes underlying irritability in premanifest Huntington’s disease. Hum Brain Mapp. 2015;36:2681–2690. 25858294 10.1002/hbm.22799 PMC6869704

[49] Perry DC , Whitwell JL, Boeve BF, et al Voxel-based morphometry in patients with obsessive-compulsive behaviors in behavioral variant frontotemporal dementia. Eur J Neurol. 2012;19:911–917. 22284815 10.1111/j.1468-1331.2011.03656.x PMC3351534

[50] Rosso SM , Roks G, Stevens M, et al Complex compulsive behaviour in the temporal variant of frontotemporal dementia. J Neurol. 2001;248:965–970. 11757960 10.1007/s004150170049

[51] Gu BM , Park JY, Kang DH, et al Neural correlates of cognitive inflexibility during task-switching in obsessive-compulsive disorder. Brain. 2008;131:155–164. 18065438 10.1093/brain/awm277

[52] Watson P , van Wingen G, de Wit S. Conflicted between goal-directed and habitual control, an fMRI investigation. eNeuro. 2018;5:ENEURO.0240-18.2018. 10.1523/ENEURO.0240-18.2018 PMC6179575 30310863

[53] Whitwell JL , Sampson EL, Loy CT, et al VBM signatures of abnormal eating behaviours in frontotemporal lobar degeneration. NeuroImage. 2007;35:207–213. 17240166 10.1016/j.neuroimage.2006.12.006

[54] Perry DC , Sturm VE, Seeley WW, Miller BL, Kramer JH, Rosen HJ. Anatomical correlates of reward-seeking behaviours in behavioural variant frontotemporal dementia. Brain. 2014;137:1621–1626. 24740987 10.1093/brain/awu075 PMC4032100

[55] Woolley JD , Gorno-Tempini ML, Seeley WW, et al Binge eating is associated with right orbitofrontal-insular-striatal atrophy in frontotemporal dementia. Neurology. 2007;69:1424–1433. 17909155 10.1212/01.wnl.0000277461.06713.23

[56] Finney CM , Mendez MF. Diogenes syndrome in frontotemporal dementia. Am J Alzheimers Dis Demen. 2017;32:438–443. 10.1177/1533317517717012 PMC10852732 28660777

[57] Naasan G , Shdo SM, Rodriguez EM, et al Psychosis in neurodegenerative disease: Differential patterns of hallucination and delusion symptoms. Brain. 2021;144:999–1012. 33501939 10.1093/brain/awaa413 PMC8041322

[58] Shinagawa S , Nakajima S, Plitman E, et al Psychosis in frontotemporal dementia. J Alzheimers Dis. 2014;42:485–499. 24898651 10.3233/JAD-140312

[59] Allen P , Larøi F, McGuire PK, Aleman A. The hallucinating brain: A review of structural and functional neuroimaging studies of hallucinations. Neurosci Biobehav Rev. 2008;32:175–191. 17884165 10.1016/j.neubiorev.2007.07.012

[60] Alderson-Day B , McCarthy-Jones S, Fernyhough C. Hearing voices in the resting brain: A review of intrinsic functional connectivity research on auditory verbal hallucinations. Neurosci Biobehav Rev. 2015;55:78–87. 25956256 10.1016/j.neubiorev.2015.04.016 PMC5901708

[61] Sellami L , Bocchetta M, Masellis M, et al Distinct neuroanatomical correlates of neuropsychiatric symptoms in the three main forms of genetic frontotemporal dementia in the GENFI cohort. J Alzheimers Dis. 2018;65:147–163. 30010122 10.3233/JAD-180053 PMC6087430

[62] Devenney EM , Landin-Romero R, Irish M, et al The neural correlates and clinical characteristics of psychosis in the frontotemporal dementia continuum and the C9orf72 expansion. NeuroImage Clin. 2017;13:439–445. 28116236 10.1016/j.nicl.2016.11.028 PMC5233794

[63] Waldö M L , Gustafson L, Passant U, Englund E. Psychotic symptoms in frontotemporal dementia: A diagnostic dilemma? Int Psychogeriatr. 2015;27:531–539. 25486967 10.1017/S1041610214002580 PMC4413855

[64] Palaniyappan L , Liddle PF. Does the salience network play a cardinal role in psychosis? An emerging hypothesis of insular dysfunction. J Psychiatry Neurosci. 2012;37:17–27. 21693094 10.1503/jpn.100176 PMC3244495

[65] Benson D . The Geschwind syndrome. Adv Neurol. 1991;55:411–421. 2003418

[66] Postiglione A , Milan G, Pappatà S, et al Fronto-temporal dementia presenting as Geschwind’s syndrome. Neurocase. 2008;14:264–270. 18704833 10.1080/13554790802269976

[67] Roberts J , Robertson M, Trimble M. The lateralising significance of hypergraphia in temporal lobe epilepsy. J Neurol Neurosurg Amp Psychiatry. 1982;45:131–138. 10.1136/jnnp.45.2.131 PMC1083040 7069424

[68] Seeley WW , Bauer AM, Miller BL, et al The natural history of temporal variant frontotemporal dementia. Neurology. 2005;64:1384–1390. 15851728 10.1212/01.WNL.0000158425.46019.5C PMC2376750

[69] Veronelli L , Makaretz SJ, Quimby M, Dickerson BC, Collins JA. Geschwind syndrome in frontotemporal lobar degeneration: Neuroanatomical and neuropsychological features over 9 years. Cortex. 2017;94:27–38. 28711815 10.1016/j.cortex.2017.06.003 PMC5565695

[70] Waxman SG , Geschwind N. Hypergraphia in temporal lobe epilepsy. Neurology. 1974;24:629–629. 4209727 10.1212/wnl.24.7.629

[71] Okamura T , Fukai M, Yamadori A, Hidari M, Asaba H, Sakai T. A clinical study of hypergraphia in epilepsy. J Neurol Neurosurg Amp Psychiatry. 1993;56:556–559. 10.1136/jnnp.56.5.556 PMC1015019 8505651

[72] Wu TQ , Miller ZA, Adhimoolam B, et al Verbal creativity in semantic variant primary progressive aphasia. Neurocase. 2015;21:73–78. 24329034 10.1080/13554794.2013.860179 PMC4284199

