# Supplementary material for: Behavioural subphenotypes and their anatomic correlates in neurodegenerative disease
Source: Brain Commun. 2023 Feb 27;5(2):fcad038. doi: 10.1093/braincomms/fcad038 (PMC9999361; doi:10.1093/braincomms/fcad038)
Supplement: fcad038_Supplementary_Data [file fcad038_supplementary_data.pdf]

Supplementary Material

Supplementary Figure 1. VBM analyses with each component controlling for diagnosis

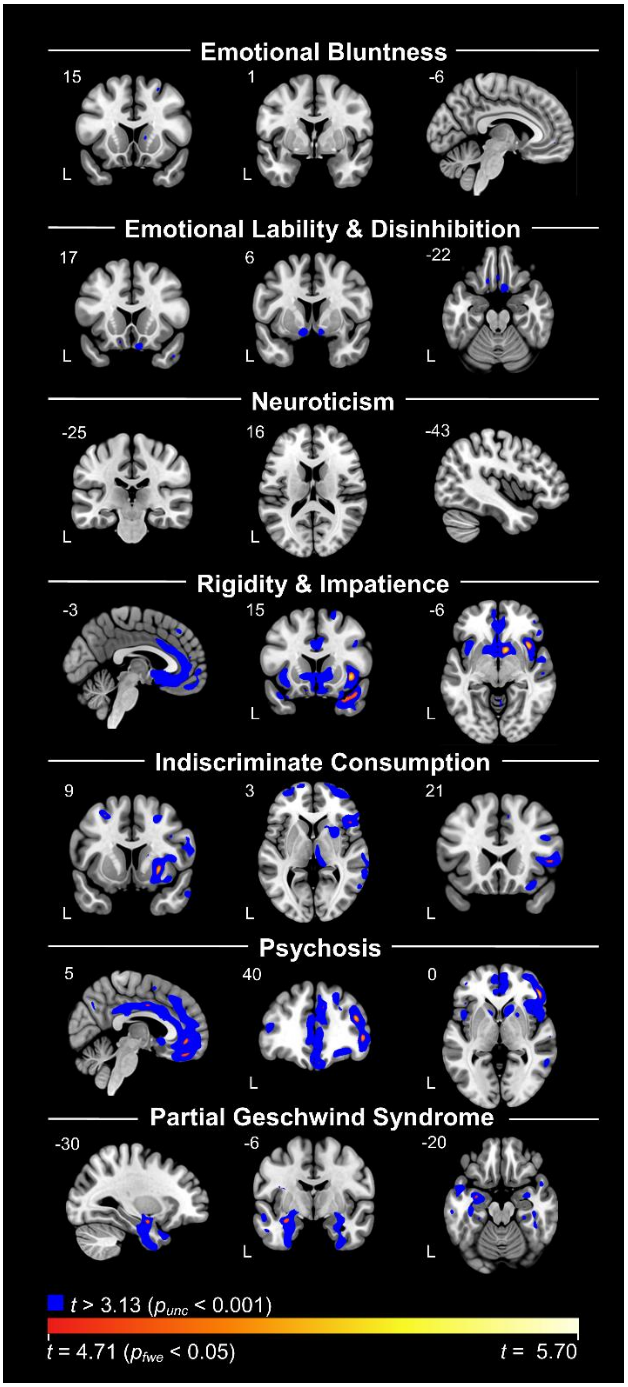

Blue represents results at uncorrected $p < .001$ levels. Red/yellow/white spectrum represents findings that survived FWE corrections at $p < .05$. Components 1, 2 and 3 did not have any findings at corrected levels. Each analysis controlled for total intracranial volume, age, sex, and scanner type (dummy coded), and diagnosis (dummy coded).

Supplementary Figure 2. Atrophy of diagnostic groups compared to healthy controls

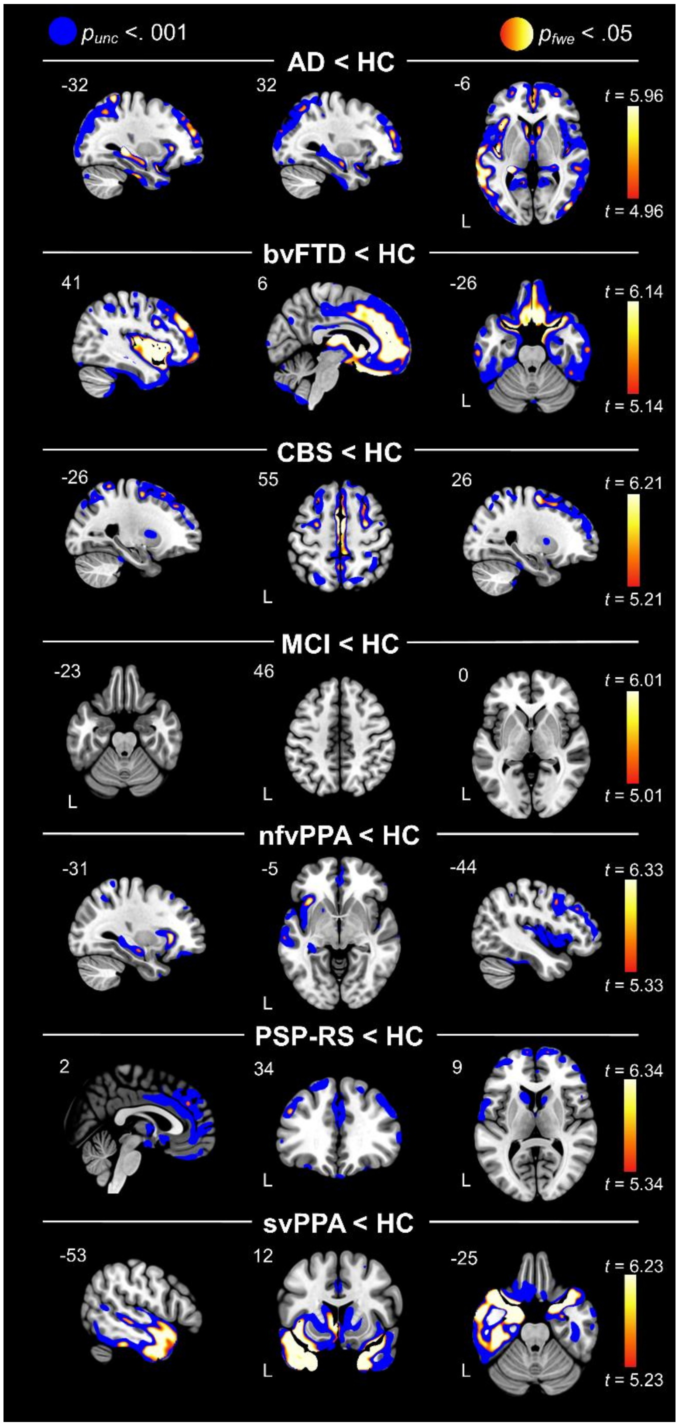

Clusters represent areas of greater volume loss in dementia groups compared to healthy controls. Blue represents results at uncorrected $p < .001$ levels. Red/yellow/white spectrum represents findings that survived FWE corrections at $p < .05$. No clusters survived at corrected and uncorrected levels for the comparison between MCI and HC. Each analysis controlled for total intracranial volume, age, sex, and scanner type (dummy coded).

Supplementary Figure 3. Scree plot of the principal component analysis

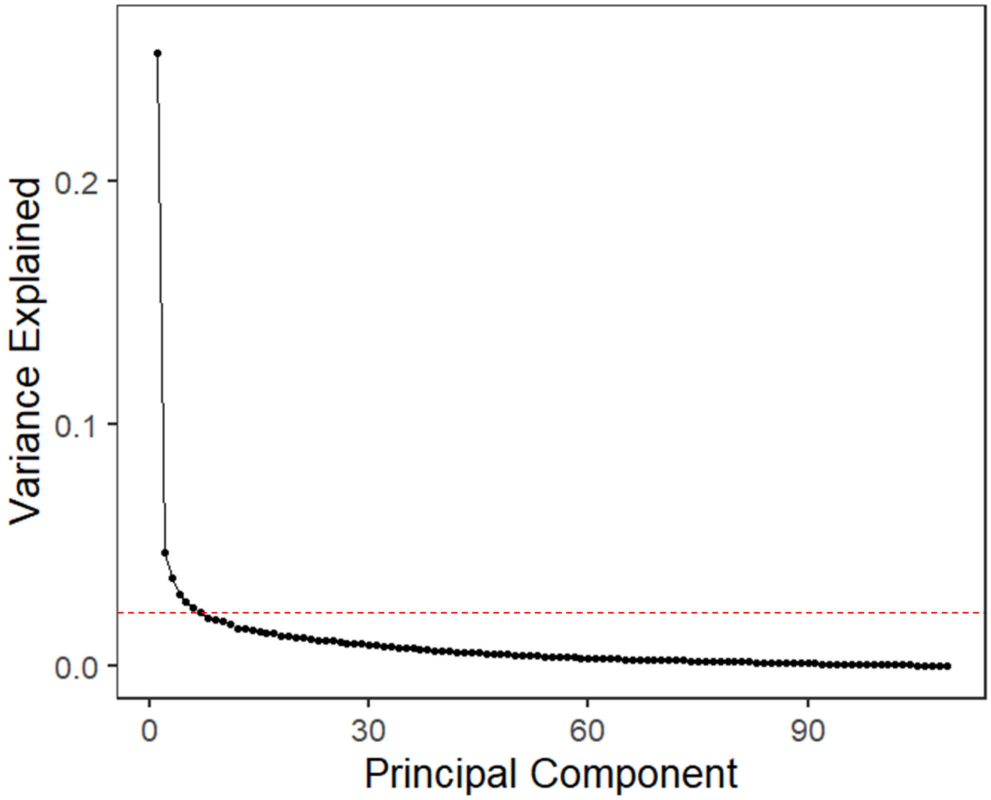

The dotted red line represents the seven-component cut off point. The total variance explained from the seven-components was 44 percent.

Supplementary Figure 4. Boxplots for each component across combined diagnostic groups

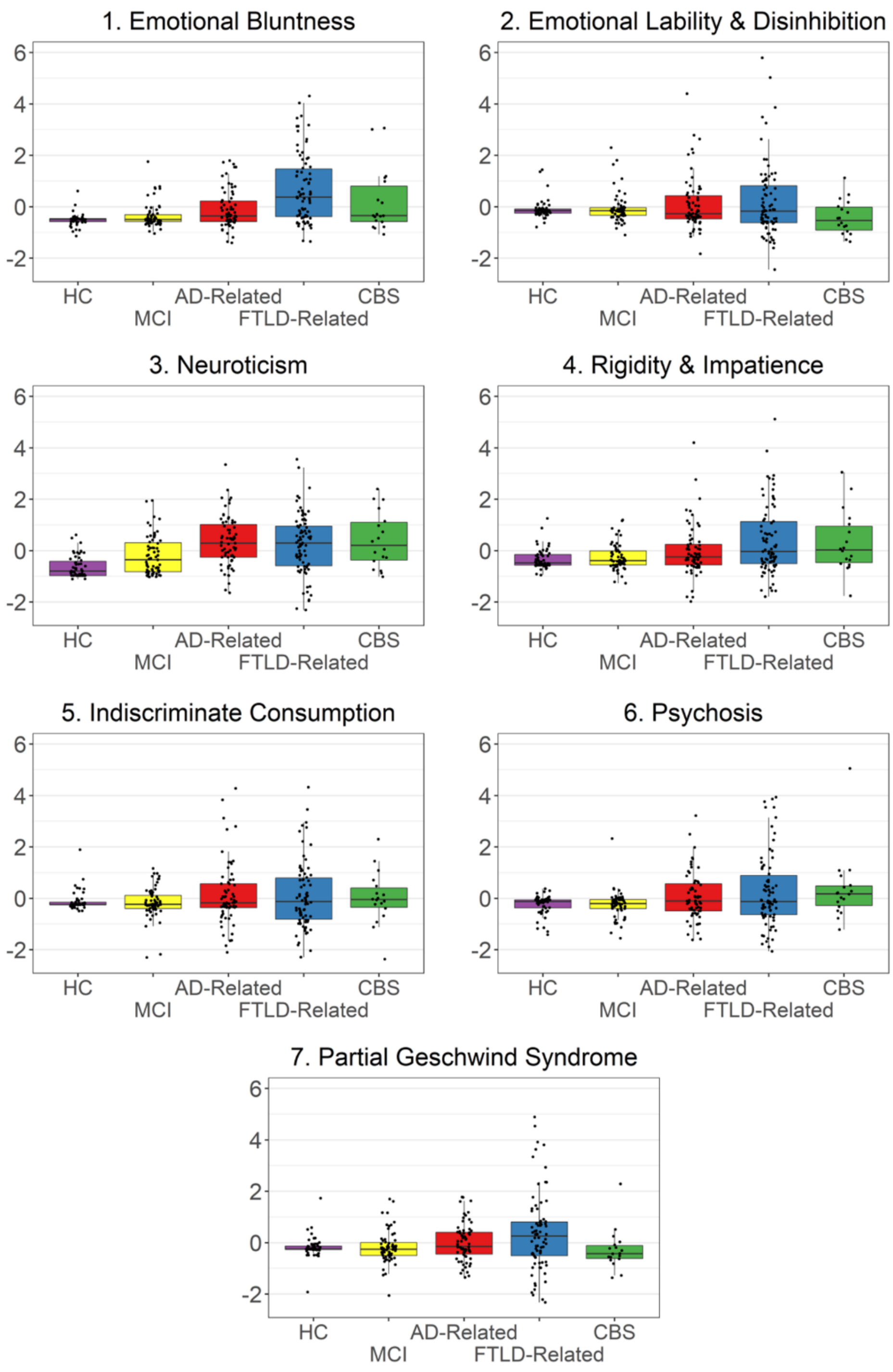

AD-Related combines the Alzheimer's disease and logogenic variant primary progressive aphasia diagnoses. FTLD-Related combines behavioral variant frontotemporal dementia, nonfluent variant primary progressive aphasia, semantic variant primary progressive aphasia and progressive supranuclear palsy-Richardson syndrome groups.

Supplementary Table 1. Anatomical correlates of component scores controlling for diagnosis

Component	Anatomical Region	Cluster Extent (mm^3)	Peak MNI Coordinates x	y	z	Max T
Comp. 1						
	—	—	—	—	—	—
Comp. 2						
	—	—	—	—	—	—
Comp. 3						
	—	—	—	—	—	—
Comp. 4						
	Caudate, R	85	9	11	-8	5.52
	Insula, R	147	38	17	-5	5.42
	Temporal Pole Superior, R	217	44	9	-23	5.32
	Temporal Middle, R	39	57	-3	-21	5.23
Comp. 5						
	Putamen, R	52	27	9	-3	5.25
	Frontal Superior, 2 R	27	29	56	11	5.03
	Frontal Inferior Triangularis, R	15	48	21	5	4.86
Comp. 6						
	Frontal Inferior Triangularis, R	131	50	36	-2	5.36
	Frontal Middle 2, R	71	41	41	21	5.27
	Frontal Inferior Operculum, R	49	54	18	12	5.24
	Frontal Medial Orbital, R	38	6	45	-6	5.05
	Cingulate Middle, R	16	9	38	33	4.97
	Rectus, R	30	3	45	-23	4.95
	OFC anterior, R	15	24	45	-14	4.95
	Cingulate Middle, R	11	5	-2	38	4.85
Comp. 7						
	Amygdala, L	31	-30	-6	-17	4.99

All clusters presented are $p_{FWE} < .05$. Minimum extent = 10. Table shows all local maxima separated by more than 20 mm. Regions were labeled using the AAL2 atlas. x, y, and z = Montreal Neurological Institute (MNI) coordinates in the, left-right, anterior-posterior, and inferior-superior dimensions, respectively. All contrasts are negative (atrophy related to higher component scores). No clusters survived for positive contrasts. OFC = orbitofrontal cortex.

Supplementary Table 2. Loadings from Principal Component Analysis

Manchester Item	Comp. 1: "Emotional Bluntness"	Comp. 2: "Emotional Lability & Disinhibition"	Comp. 3: "Neuroticism"	Comp. 4: "Rigidity & Impatience"	Comp. 5: "Indiscriminate Consumption"	Comp. 6: "Psychosis"	Comp. 7: "Partial Geschwind Syndrome"
1. Loss of embarrassment	0.51	0.29	0.06	0.32	0.43	-0.06	0.23
2. Breaks social norms	0.38	0.39	0.03	0.20	0.42	-0.05	0.20
3. Social inattention	0.03	0.47	0.07	0.32	0.20	-0.05	0.26
4. Dogmatism	0.08	0.37	0.29	0.12	0.27	-0.01	0.24
5. Overly literal	0.20	0.33	0.23	0.09	0.15	-0.03	0.12
6. Excessive staring	0.28	0.24	0.04	0.11	0.27	0.38	0.10
7. Reduced eye contact	0.49	0.05	0.27	0.11	0.01	0.18	0.02
8. Lacks Insight	0.48	0.23	0.22	0.28	0.34	0.01	0.26
9. Unselfconscious	0.60	0.25	-0.06	0.23	0.31	0.01	0.22
10. Self-Deprecatory	0.03	0.13	0.45	0.01	-0.16	-0.03	-0.06
11. Selfishness	0.37	0.40	0.21	0.19	0.31	0.02	0.20
12. Loss of empathy	0.61	0.41	0.09	0.07	0.24	0.03	0.27
13. Cruelty	0.16	0.38	0.11	-0.03	0.40	0.19	-0.02
14. Social avoidance	0.38	-0.05	0.56	-0.08	0.04	0.21	0.05
15. Seeks out social contact	0.02	0.25	0.03	0.45	0.20	-0.12	0.25
16. Decreased affection	0.51	0.15	0.27	-0.06	0.07	0.12	0.06
17. Increased affection	0.01	0.03	0.07	0.31	0.22	-0.02	-0.02
18a. Reduced emotional display: Happiness	0.75	0.18	0.30	0.12	-0.01	0.05	0.08
18b. Reduced emotional display: Sadness	0.81	0.25	0.10	0.17	0.10	0.13	0.12
18c. Reduced emotional display: Fear	0.74	0.21	0.09	0.21	0.04	0.04	0.11
18d. Reduced emotional display: Anger	0.76	0.11	0.07	0.01	0.09	0.03	0.05
18e. Reduced emotional display: Surprise	0.83	0.20	0.02	0.13	0.03	0.13	0.01
18f. Reduced emotional display: Disgust	0.84	0.16	0.00	0.07	0.13	-0.06	0.03
19a. Exaggerated emotional display: Happiness	0.11	0.61	0.07	0.14	-0.02	0.01	-0.03
19b. Exaggerated emotional display: Sadness	0.10	0.46	0.36	0.12	-0.11	0.10	-0.15
19c. Exaggerated emotional display: Fear	0.18	0.49	0.35	0.02	-0.06	0.04	-0.13
19d. Exaggerated emotional display: Anger	0.11	0.60	0.45	0.09	0.18	-0.02	-0.02
19e. Exaggerated emotional display: Surprise	0.19	0.72	0.16	0.08	0.02	0.02	-0.07

19f. Exaggerated emotional display: Disgust	0.09	0.69	0.21	0.03	0.08	-0.01	-0.05
20. Loss of humor	0.69	0.15	0.23	0.12	0.07	-0.01	0.27
21. Childish humor	0.12	0.43	0.07	0.28	0.17	0.15	0.01
22. Childish mannerisms	0.25	0.43	-0.06	0.33	0.11	0.14	0.14
23. Gulliblility	0.47	0.33	0.06	0.20	0.28	-0.03	0.25
24. Disinhibition	0.23	0.52	0.06	0.29	0.32	0.00	0.10
25. Bravado	0.11	0.35	0.21	0.08	0.25	0.03	0.18
26. Neglect of hygiene	0.62	0.07	0.15	0.12	0.40	0.15	0.13
27. Neglect of table manners	0.61	0.05	0.18	0.25	0.40	0.17	0.10
28. Reactivity	0.22	0.26	0.61	0.07	0.10	-0.02	0.00
29. Excessive worrying	0.10	0.21	0.55	0.26	0.00	0.13	0.01
30. Loss of interest	0.52	0.17	0.38	0.17	0.18	0.25	-0.01
31. Irritability	0.08	0.37	0.54	0.10	0.14	0.03	0.01
32. Aggression	0.05	0.43	0.38	0.15	0.28	-0.14	0.07
33. Struggles with multi-tasking	0.36	0.08	0.48	0.17	0.17	0.18	-0.01
34. Loss of awareness of pain	0.46	0.10	0.05	-0.05	0.23	0.08	-0.03
35. Hypochondriasis	0.08	0.33	0.13	0.15	-0.07	0.21	0.09
36. Loss of smell	0.35	-0.08	0.16	-0.01	0.28	-0.08	0.09
37. Exaggerated sensory response	0.28	0.34	0.16	0.18	0.06	0.11	0.10
38. Exaggerated heat/cold response	0.22	0.25	0.18	0.13	-0.01	0.20	0.14
39. Weather tolerance	0.15	0.03	0.11	0.03	0.18	0.09	0.14
40. Preference for sweet foods	0.40	0.15	0.14	0.26	0.28	0.12	0.11
41. Preference for savory foods	0.30	0.15	0.08	0.23	0.20	0.07	-0.21
42. Preference for color of food	0.18	0.25	0.05	0.21	-0.05	0.02	-0.19
43. Food fads	0.18	0.19	0.28	0.25	0.04	-0.02	0.06
44. Loss of discrimination	0.50	-0.02	0.05	0.20	0.43	0.07	0.02
45. Overeats	0.36	0.15	0.02	0.12	0.52	0.09	-0.20
46. Eats continually if food present	0.43	0.06	0.00	0.16	0.48	0.13	-0.21
47. Steals food from others’ plates	0.40	0.09	-0.03	0.00	0.50	-0.06	-0.09
48.Seeks out food	0.30	0.16	0.14	0.23	0.54	0.06	-0.14
49. Crams food	0.25	0.01	0.09	0.29	0.15	0.07	-0.03
50. Weight gain/loss	0.41	0.02	0.18	0.23	0.25	-0.01	-0.15
51. Increased alcohol consumption	0.04	0.16	0.15	0.01	0.22	0.03	-0.05
53. Oral exploration of objects	0.28	0.11	0.00	-0.03	0.13	-0.06	0.05

54. Wandering	0.12	0.07	0.02	0.29	0.36	0.08	0.10
55. Motor restlessness	0.24	0.27	0.25	0.47	0.27	0.23	-0.01
56. Hyposexuality	0.43	0.00	0.31	0.03	0.01	0.08	0.12
57. Hypersexuality	0.24	0.05	-0.01	0.28	0.21	0.08	-0.09
58. Hyposomnia	0.09	0.03	0.32	0.07	0.08	0.05	-0.03
59. Hypersomnia	0.40	0.04	0.13	0.15	0.03	0.12	0.05
60. Daytime hypersomnia	0.47	0.03	0.22	0.17	0.12	0.02	0.00
61. Acts out dreams	0.08	-0.04	0.28	0.28	0.17	0.06	-0.02
62. Simple motor stereotypes	0.46	0.17	0.18	0.31	0.12	0.36	0.20
63. Complex motor routines	0.06	0.07	0.06	0.35	0.00	-0.01	-0.04
64. Paces fixed route	0.20	0.34	-0.11	0.21	0.17	0.34	-0.08
65. Verbal stereotypes	0.32	0.30	-0.03	0.27	0.20	0.06	0.26
66. Verbal perseverations	0.15	0.30	0.04	0.35	0.13	0.27	0.15
67. Repetitive themes	0.26	0.23	0.21	0.36	0.28	0.08	0.27
68. Needs to do things immediately	0.20	0.16	0.41	0.51	0.11	0.09	0.20
69. Rigidity	0.21	0.19	0.42	0.46	0.17	0.03	0.31
70. Hypergraphia	0.03	0.14	0.18	0.12	0.06	0.11	0.42
71. Adherence to daily routine	0.17	0.19	0.37	0.35	0.02	0.00	0.16
72. Counts objects	0.15	0.24	-0.04	0.36	0.01	-0.06	0.31
73. Aligns/Arranges	0.13	0.15	0.09	0.45	0.10	0.21	0.12
74. Overconcern with cleanliness	0.05	0.09	-0.01	0.39	-0.02	0.29	0.14
75. Poor organization	0.22	0.15	0.30	0.01	0.24	0.12	0.03
76. Excessive attention to detail	0.06	0.05	0.15	0.46	0.01	-0.02	0.28
77. Excessive checking	0.06	0.24	0.18	0.37	-0.03	0.41	0.13
78b. Compulsive gaming: Card games	0.13	-0.03	-0.02	0.08	-0.02	0.07	0.38
78c. Compulsive gaming: Games w/shapes	0.13	-0.01	-0.11	0.09	-0.12	0.01	0.43
79. Petty theft	0.23	0.09	-0.09	0.05	0.27	-0.04	0.31
80. Defies authority	0.20	0.42	0.13	0.01	0.43	-0.04	0.20
81. Changes in purchasing habits	0.22	0.26	0.13	0.04	0.25	0.25	0.08
82. Unusual toileting routines	0.31	0.18	-0.02	0.42	0.21	0.07	0.04
85. Artistic Abilities	-0.03	0.12	0.04	0.20	-0.13	-0.14	-0.08
86. Hoarding	0.21	0.15	0.13	0.12	0.53	0.15	0.26
87. Touches or handles objects	0.14	0.05	0.01	0.30	0.54	0.27	0.07
88. Echolalia	0.15	0.37	0.01	-0.01	0.27	0.32	0.08
89. Echopraxia	0.26	0.23	-0.13	0.03	0.18	0.14	0.19

91. Reads notices aloud	0.13	0.41	0.02	0.27	0.19	0.15	0.16
92. Suspiciousness	0.10	0.39	0.43	0.01	0.17	0.29	0.18
93. Delusions of theft	0.04	0.25	0.25	-0.11	0.17	0.45	0.35
95. Misidentification phenomena	0.01	0.30	0.02	0.17	0.12	0.12	0.15
96. Visual hallucinations/illusions	0.08	-0.05	0.00	0.06	0.00	0.49	-0.02
97. Auditory hallucinations/illusions	0.01	0.03	0.17	-0.01	0.10	0.48	-0.03

Values represent loadings for each question on each of the seven components.

Supplementary Table 3. ANCOVA and post-hoc statistics for comparisons in Figure 1

Component	ANCOVA Results	Significant Post-hoc Comparisons		
		Pairwise Group	T-Statistic	P-Value
Comp. 1				
	F(8, 270) = 13.32, *p* < .001			
		bvFTD - AD	6.40	< 0.001
		svPPA - AD	3.76	< 0.01
		CBS - bvFTD	-3.60	< 0.01
		lvPPA - bvFTD	-3.51	0.01
		MCI - bvFTD	-7.79	< 0.001
		HC - bvFTD	-8.44	< 0.001
		nfvPPA - bvFTD	-5.04	< 0.001
		PSP-RS - MCI	3.13	0.04
		svPPA - MCI	5.00	< 0.001
		PSP-RS - HC	3.62	< 0.01
		svPPA - HC	5.59	< 0.001
		svPPA - nfvPPA	3.52	0.01
Comp. 2				
	F(8, 270) = 4.07, *p* < .001			
		bvFTD - AD	3.23	0.03
		CBS - bvFTD	-3.85	< 0.01
		MCI - bvFTD	-3.47	0.01
		HC - bvFTD	-3.60	< 0.01
		PSP-RS - bvFTD	-3.86	< 0.01
		PSP-RS - lvPPA	-3.14	0.04
Comp. 3				
	F(8, 270) = 8.57, *p* < .001			
		MCI - AD	-3.54	0.01
		HC - AD	-6.03	< 0.001
		PSP-RS - bvFTD	4.36	< 0.001
		HC - CBS	-4.23	< 0.001
		PSP-RS - MCI	4.92	< 0.001
		nfvPPA - HC	3.50	0.01
Comp. 4				
	F(8, 270) = 3.90, *p* < .001			
		MCI - bvFTD	-3.15	0.04
		HC - bvFTD	-3.48	0.01
		svPPA - MCI	3.85	< 0.01
		svPPA - HC	4.13	< 0.01
Comp. 5				

	$F(8, 270) = 3.26, p < .01$			
		MCI - bvFTD	-3.96	< 0.01
		HC - bvFTD	-3.74	< 0.01
		svPPA - bvFTD	-4.21	< 0.001
Comp. 6				
	$F(8, 270) = 3.58, p < .01$			
		MCI - bvFTD	-3.27	0.03
		HC - bvFTD	-3.18	0.04
		PSP-RS - MCI	3.78	< 0.01
		PSP-RS - HC	3.71	< 0.01
		svPPA - PSP-RS	-3.27	0.03
Comp. 7				
	$F(8, 270) = 9.84, p < .001$			
		svPPA - AD	7.414	< 0.001
		svPPA - bvFTD	6.77	< 0.001
		svPPA - CBS	6.615	< 0.001
		svPPA - lvPPA	3.289	0.03
		svPPA - MCI	8.124	< 0.001
		svPPA - HC	7.879	< 0.001
		svPPA - nfvPPA	5.094	< 0.001
		svPPA - PSP-RS	5.979	< 0.001

ANCOVA analyses included age and sex as nuisance covariates. Tukey post-hoc tests were performed to determine significant pairwise differences. ANCOVA = Analysis of covariance; bvFTD = behavioral variant frontotemporal dementia; AD = Alzheimer’s disease; CBS = corticobasal syndrome; PSP-RS = progressive supranuclear palsy-Richardson syndrome; nfvPPA = nonfluent variant primary progressive aphasia; svPPA = semantic variant primary progressive aphasia; MCI = mild cognitive impairment; lvPPA = logopenic variant primary progressive aphasia; HC = healthy control.
